# Loss of receptor tyrosine kinase-like orphan receptor 2 impairs the osteogenesis of mBMSCs by inhibiting signal transducer and activator of transcription 3

**DOI:** 10.1186/s13287-020-01646-2

**Published:** 2020-03-26

**Authors:** Lizhen Lei, Zhuwei Huang, Jingyi Feng, Zijing Huang, Yiwei Tao, Xiaoli Hu, Xiaolei Zhang

**Affiliations:** 1 Guangdong Province Key Laboratory of Stomatology, Guangzhou, 510080 Guangdong China; 2 Department of Operative Dentistry and Endodontics, Guanghua School and Hospital of Stomatology, Sun Yat-sen University, Guangzhou, 510055 Guangdong China

**Keywords:** Ror2, Stat3, BMSCs, Osteogenesis

## Abstract

**Background:**

Receptor tyrosine kinase-like orphan receptor 2 (Ror2) plays a key role in bone formation, but its signaling pathway is not completely understood. Signal transducer and activator of transcription 3 (Stat3) takes part in maintaining bone homeostasis. The aim of this study is to reveal the role and mechanism of Ror2 in the osteogenic differentiation from mouse bone marrow mesenchymal stem cells (mBMSCs) and to explore the effect of Stat3 on Ror2-mediated osteogenesis.

**Methods:**

*Ror2* CKO mice were generated via the Cre-loxp recombination system using Prrx1-Cre transgenic mice. Quantitative real-time PCR and western blot were performed to assess the expression of Stat3 and osteogenic markers in Ror2-knockdown mBMSCs (mBMSC-sh-Ror2). After being incubated in osteogenic induction medium for 3 weeks, Alizarin Red staining and western blot were used to examine the calcium deposit and osteogenic markers in Stat3 overexpression in mBMSC-sh-Ror2.

**Results:**

Loss of Ror2 in mesenchymal or osteoblast progenitor cells led to a dwarfism phenotype in vivo. The mRNA expression of osteogenic markers (osteocalcin, osteopontin (OPN), and collagen I) in the ulna proximal epiphysis of *Ror2* CKO mice was significantly decreased (*P* < 0.05). The mRNA and protein expression of Stat3 and osteogenic markers (Runx2, osterix, and OPN) decreased in mBMSC-sh-Ror2 cells (*P* < 0.05). The overexpression of Stat3 in mBMSC-sh-Ror2 cells rescued the calcium deposit and expression of Runx2, osterix, and OPN to a level comparable to normal mBMSCs.

**Conclusions:**

Ror2 was essential for skeleton development by regulating mBMSCs’ osteogenesis and osteoblast differentiation. Loss of Ror2 may impair the osteogenesis of mBMSCs by inhibiting Stat3.

## Background

The proliferation and differentiation of mesenchymal stem cells toward osteoblasts is important for bone development and formation. The process involves in multiple regulatory signaling pathways and transcription factors [1, 2]. Both canonical and non-canonical Wnt signaling pathways play essential roles in mesenchymal stem cells differentiation into osteoblasts along with their proliferation and mineralization [1, 3, 4].

The extracellular region of receptor tyrosine kinase-like orphan receptor 2 (Ror2) contains a cysteine-rich domain (CRD), which exhibits similarities to the CRDs found in the Frizzled (Fzd) family of seven transmembrane Wnt receptors [5–7]. Previous studies have identified Ror2 functions as a Wnt receptor/coreceptor regulated by canonical and non-canonical Wnt signaling pathways [8–12]. Mice with Ror2 homozygous mutation displayed shortened mandible, defects of derivatives of Meckel’s cartilage, and notably shortening of the long bones of the appendicular skeleton [13, 14]. Ror2 mutation in humans accounts for recessive Robinow syndrome, which is characterized by macrocephaly, short stature, and mesomelic limb shortening, mostly in the forearms and brachydactyly [15, 16]. The severe skeletal phenotypes in both Ror2 mutation mice and Robinow patients suggested a significant role of Ror2 in bone development and formation [11, 13–16]. Available in vitro studies have proven that Ror2 can promote the osteogenic differentiation of mesenchymal stem cells [3, 17, 18]. Nevertheless, direct evidence demonstrating the participation of Ror2 in osteogenesis of osteoblastic cell lineages in vivo is still required. The Ror2-mediated signaling pathways in osteogenesis of osteoblastic cell lineages are not completely understood. The Prrx1-Cre mice express Cre recombinase under the regulation of the paired related homeobox gene-1(Prx1)-derived regulatory element [19, 20]. When Prrx1-Cre transgenic mice are crossed with a strain containing a loxp site-flanked sequence of interest, Cre-mediated recombination results in deletion of the floxed sequence in all mesenchyme-derived cells in the limbs and craniofacial tissue [19–22]. Therefore, in order to elucidate the role of Ror2 in osteogenesis of osteoblastic cell lineages in vivo, Ror2^f/f^ transgenic mice which possess loxp sites flanking exons 3–4 of Ror2 gene [23] and the Prrx1-Cre transgenic mice were used to generate mice with Ror2 gene-specific deletion in mesenchymal progenitors.

The *cytoplasmic* transcription factor signal transducer and activator of transcription 3 (Stat3) activated by various cytokines and growth factors participated in cell proliferation, differentiation, and apoptosis [24, 25]. Activated Stat3 proteins translocated into the nucleus and then exerted transcriptional functions, thus taking part in tumorigenesis, self-renewal, and pluripotency of stem cells [26]. Mice with selective disruption of Stat3 in osteoblasts or osteocytes showed reduced bone formation [27, 28]. High level of Ror2 expression in platinum-resistant ovarian tumor was found to be coupled with upregulation of Stat3, suggesting that Stat3 might act as the signaling downstream of Ror2 [29]. IL-6 induced the transcription of Ror2 to accelerate osteoblast-like differentiation and calcification in human adipose tissue-derived mesenchymal stem cell (hADSCs), while suppression of Stat3 in hADSCs inhibited IL-6-mediated Ror2 expression and mineralization [30]. However, the role of Stat3 in Ror2-mediated osteogenesis of mBMSCs has not been unveiled.

In this study, we investigated the role of Ror2 in embryonic and neonatal bone formation though transgenic mice of limb bud mesenchyme and craniofacial mesenchyme-specific Ror2 deficiency. In addition, the effect of Stat3 on Ror2-mediated osteogenesis of mBMSCs was explored in vitro.

## Methods

### Mouse lines

**Fig. 1:**
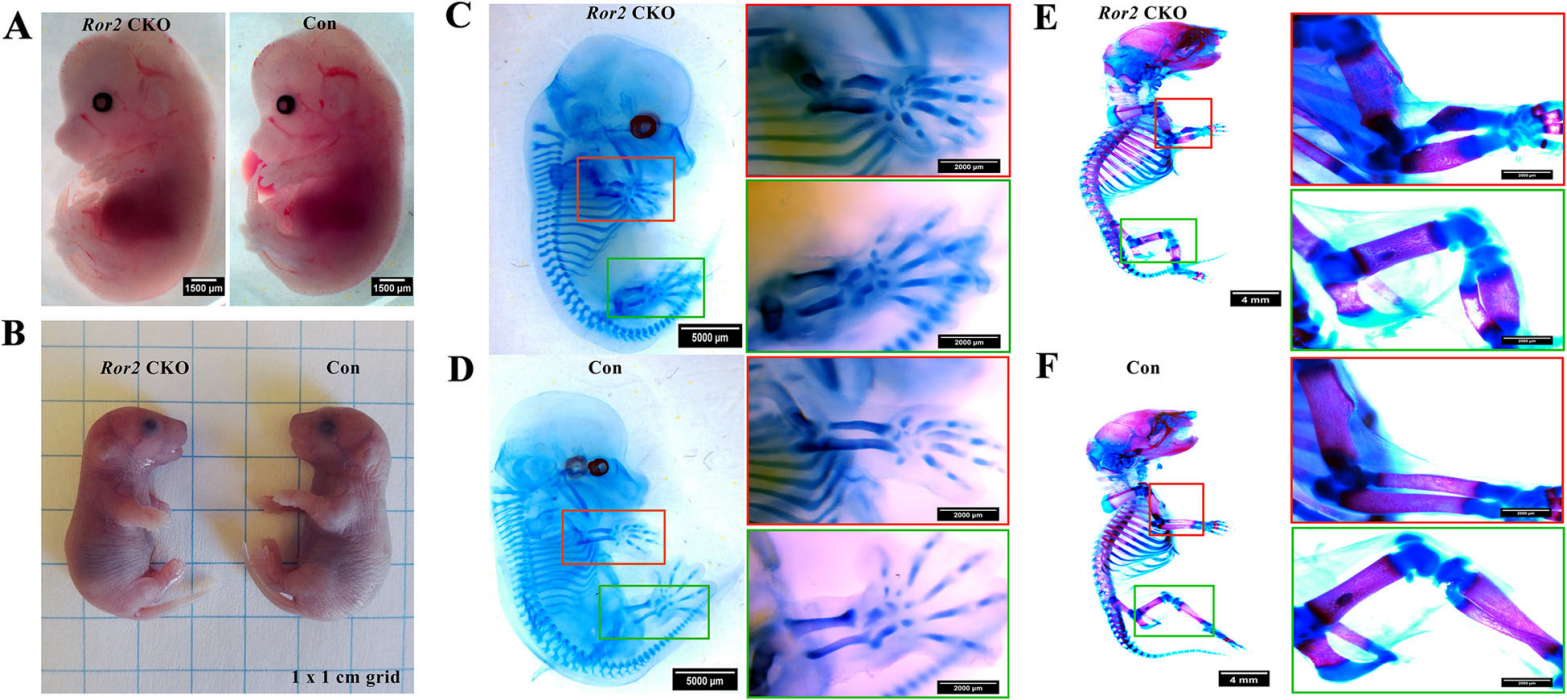
The dwarfism phenotype of *Ror2* CKO mice. Compared with the littermate control (Con) mice, the limbs were significantly shortened in Prrx1-Cre; Ror2^f/f^ (*Ror2* CKO) mice at both embryonic (E14.5) and neonatal (P0) stage. **a**, **b** Ex vivo observation of *Ror2* CKO and control mice at E14.5 (**a**) and P0 (**b**), scale bar = 1500 μm. **c**–**f** Whole-mount skeletal staining of *Ror2* CKO and Con mice at E14.5 (**c**, **d**), scale bar = 5000 μm, and P0 (**e**, **f**), scale bar = 4 mm, respectively. The boxed region is enlarged in the right panel, scale bar = 2000 μm

Mice were used according to federal guidelines and as approved by the Animal Ethical and Welfare Committee of Sun Yat-sen University (approval number SYSU-IACUC-2018-000275). Mice were described in the literature and purchased from the Jackson Laboratory: Prrx1-Cre (stock no. 005584) and Ror2^f/f^ (stock no. 018354) [20, 23]. To generate Ror2 conditional knockout mice, Ror2^f/f^ mice were first crossed with Prrx1-Cre transgenic mice to obtain Prrx1-Cre; Ror2^f/+^ mice. Then, Prrx1-Cre; Ror2^f/+^ mice were crossed with Ror2^f/f^ mice to get Prrx1-Cre; Ror2^f/f^ (hereafter called *Ror2* CKO) mice. For embryos or neonatal mice, both male and female were used in the analyses as sex could not be clearly identified in embryos or neonatal mice. Three or more littermate mice per group were examined, and representative images were shown in Fig. 1. Genotyping was conducted by polymerase chain reaction (PCR) of tail lysate. Primer sequences were provided in Table 1.

**Table 1:** Primers for PCR and qRT-PCR

Gene	Forward	Reverse	Application
Prrx1	GCG GTC TGG CAG TAA AAA CTA TC	GTG AAA CAG CAT TGC TGT CAC TT	PCR
Ror2	TGC AGG TTT TGA GCC CTA AC	CGA GAA TGA CTT CCC TGT CC	PCR
GAPDH	CCTTCCGTGTTCCTACCC	CAACCTGGTCCTCAGTGTAG	qRT-PCR
Ror2	ACGCAATGTGCTGGTGTAC	TGTCAGAGTCGATGGAGAAC	qRT-PCR
Stat3	CATCCTGAAGCTGACCCAGG	TATTGCTGCAGGTCGTTGGT	qRT-PCR
Runx2	CCGTCACCTCCATCCTCTTTC	AATACGCATCACAACAGCCACA	qRT-PCR
OSX	ACCCTTCCCTCACTCATTTCCTG	TGCCTTGTACCACGAGCCATAG	qRT-PCR
OPN	CTCCAATCGTCCCTACAGTCG	CCAAGCTATCACCTCGGCC	qRT-PCR
COL1A1	CCCAAGGAAAAGAAGCACGTC	ACATTAGGCGCAGGAAGGTCA	qRT-PCR
OCN	GCTGCCCTAAAGCCAAACTCT	AGAGGACAGGGAGGATCAAGTTC	qRT-PCR

### Skeletal staining

According to the standard protocol, skeletons from embryonic (E14.5) and neonatal (P0) mice were processed for Alizarin Red and Alcian Blue staining to visualize bone and cartilage [31].

### Histology and immunohistofluorescence (IHF) staining

Forelimbs of P0 newborn mice were isolated, fixed overnight in 4% paraformaldehyde (PFA, Servicebio, Wuhan, China), and were embedded in optimal cutting temperature (OCT) compound (Tissue-Tek). Ulna longitudinal sections (8 μm) using the elbow joint as landmark were cut and stained for both histology and IHF staining.

To assess the general histology and mineralized tissue in the region of interest, the histological stainings including hematoxylin-eosin (H&E) and Von Kossa were applied.

Using a standard protocol, IHF staining was performed with the appropriate primary antibodies and secondary antibodies (Table 2). Cryostat sections were mounted in mounting medium with DAPI from Vector Laboratories (H-1200). The images were captured by an inversion fluorescence microscope (Zeiss, Oberkochen, Germany).

**Table 2:** Antibodies used for immunoblotting

Marker (species)	Dilution	Distributor/source (catalog number)
**Primary antibody**		
RANKL mouse mAb	1:100	Novus Biologicals (12A380)
OSX rabbit mAb	1:70(IHF)	Santa Cruz (A13)
OSX rabbit mAb	1:1000(WB)	Abcam (ab22552)
OPN goat mAb	1:200(IHF)	R&D (af808)
OPN rabbit pAb	1:1000(WB)	ZEN BIO(380437)
COL1A1 goat pAb	1:200	Santa Cruz (sc-8784-R)
CD31 rabbit mAb	1:200	Biolegend (102502)
SOX9 rabbit mAb	1:300	Millipore (ab-5535)
Ror2 rabbit pAb	1:1000	CST (4105)
Stat3 mouse mAb	1:1000(WB)	CST (9139)
Stat3 mouse mAb	1:100(ICF)	CST (9139)
Runx2 rabbit mAb	1:1000	CST (8486)
pStat3 mouse mAb	1:2000	CST (4113)
**Secondary antibody**		
Anti-rabbit IgG (Fluor® 488 Conjugate)	1:500 (IHF)	Yeasen Biotech (33106ES60)
Anti-mouse IgG (Fluor® 568 Conjugate)	1:500 (IHF)	Abcam (ab175473)
Anti-rat IgG (Fluor® 594 Conjugate)	1:500 (IHF)	Yeasen Biotech (34412ES60)
Anti-mouse IgG (Fluor® 564 Conjugate)	1:300 (ICF)	Yeasen Biotech (33212ES60)
Anti-mouse IgG HRP-linked Ab	1:2000 (WB)	CST (7076)
Anti-rabbit IgG HRP-linked Ab	1:2000 (WB)	CST (7074)

*IHF* immunohistofluorescence, *WB* western blot, *ICF* immunocytofluorescence

### Cell culture

Mouse bone marrow mesenchymal stem cells (mBMSCs) isolated from the bone marrow of C57BL/6 mice were provided by Cyagen Biosciences, Inc. (Guangzhou, China). Identification of the cells according to the cell surface phenotypes and multipotency was performed by the supplier. mBMSCs were cultured in alpha-MEM (Gibco) supplemented with 10% fetal bovine serum (FBS, Gibco) and incubated at 37 °C in a humidified atmosphere of 5% CO_2_. mBMSCs in passages 6–9 were used in this study.

### Lentiviral vector construction and transduction

The recombinant lentivirus vector downregulation of mouse Ror2 gene and overexpression of mouse Stat3 gene were generated by Cyagen Biosciences, Inc. (Guangzhou, China) and iGeneBio Biotechnology (Guangzhou, China), respectively.

According to the manufacturer’s instructions, mBMSCs were infected with lentivirus-based shRNA vector downregulation of Ror2 (sh-Ror2) and lentivirus-based shRNA vector overexpression of Stat3 (sh-Stat3). Briefly, mBMSCs were plated at 1.5 × 10^4^ cells/cm^2^ in 12-well plates overnight and then infected with lentivirus in the presence of 5 μg/mL polybrene (iGeneBio Biotechnology, Guangzhou, China) for 8 h. After 72 h, mBMSCs infected with sh-Ror2 were selected with 1 μg/mL puromycin (Solarbio, Beijing, China). Those mBMSCs infected with sh-Ror2 and sh-Stat3 were selected with 1 μg/mL puromycin and 50 μg/mL hygromycin B (Solarbio, Beijing, China). The expression of Ror2 and Stat3 was assessed by quantitative real-time polymerase chain reaction (qRT-PCR) and western blot analysis.

### Osteogenic differentiation and calcium deposition determination

For osteogenic differentiation, the cells were plated at 2 × 10^4^ cells/cm^2^ in 6-well plates and cultured in 2 ml of alpha-MEM containing 10% FBS. When cells reached approximately 60–70% confluence, they were switched to the osteogenic induction medium, which consisted of alpha-MEM supplemented with 10% (vol/vol) FBS, 10^−7^ M dexamethasone (Sigma, USA), 10 mM β-glycerol phosphate (Solarbio, Beijing, China), and 50 μM ascorbate-2-phosphate (Solarbio, Beijing, China) for 3 weeks. The calcium deposition was assessed by staining with 40 mM Alizarin Red S solution (Cyagen Biosciences, Guangzhou, China) at room temperature for 15 min. The Alizarin Red S concentrations were determined by a quantitative destaining procedure using 10% cetylpyridinium chloride (CPC) (Sigma, USA) for 15 min at room temperature. The absorbance value at 562 nm was then measured by microplate spectrophotometer (Bio-Tek, UK).

### RNA isolation, reverse transcription, and qRT-PCR analysis

Total RNA of mouse forelimbs and mBMSCs was extracted using an Eastep Super total RNA extraction kit (Promega, Shanghai, China) according to the manufacturer’s instructions and then was reverse transcribed using a PrimeScriptTM RT Master Mix (Takara Bio, Ohtsu, Japan). The quantification was performed using the TB Green™ Premix Ex Taq™ II (Tli RNaseH Plus) reagent (Takara Bio, Ohtsu, Japan). The relative expression of target gene was normalized in relative to the level of GAPDH using the 2^−ΔΔCT^ method. Primer sequences were provided in the Table 1.

### Western blot

Total protein and nucleoprotein lysates from cells were extracted using the RIPA lysis buffer (CWBIOTECH, Beijing, China) according to the manufacturer’s instructions. The proteins were separated by sodium dodecyl sulfate-polyacrylamide gel electrophoresis (SDS-PAGE, Genscript, Nanjing, China) and transferred to a polyvinylidene fluoride (PVDF) membrane (Millipore, Bedford, MA, USA). The membrane was then blocked with 5% bovine serum albumin (BSA, Biofroxx, Germany) for 2 h, incubated with relevant primary antibodies (Table 2) at 4 °C overnight, and then incubated with corresponding secondary antibodies (Table 2) for 1 h at room temperature. Bands were detected with a chemiluminescence kit (Millipore, Bedford, MA, USA).

### Immunocytofluorescence (ICF) staining

Approximately 0.2 × 10^4^ cells/cm^2^ cells were seeded in 24-well plates with cover slides. When reached approximately 60% confluence, they were switched to the osteogenic induction medium for 24 h. The cells were fixed in 4% PFA for 10 min and then permeabilized in 0.1% Triton X-100 (CWBIOTECH, Beijing, China) for 5 min at room temperature. Next, the cells were blocked with 5% BSA at room temperature for 1 h and then incubated with Stat3 antibodies (Table 2) at 4 °C overnight. After a PBS wash for 15 min, the cells were incubated with corresponding secondary antibodies (Table 2) diluted in 5% BSA at room temperature for 1 h. Finally, the cells were washed for 15 min with PBS, followed by nuclear staining with DAPI (Telenbiotech, Guangzhou, China). Confocal laser scanning fluorescence microscopy (Nikon Eclipose Ni-E, Japan) was employed to analyses Stat3 staining in cells.

### Statistical analysis

The data were presented as means ± standard deviation (SD). Comparisons among groups were performed by one-way ANOVA followed by Bonferroni’s post hoc test with GraphPad Prism 7.0 software. The significance was set at *P* < .05.

## Results

### The dwarfism phenotype of *Ror2* CKO mice

The embryonic (E14.5) and neonatal (P0) *Ror2* CKO mice displayed dwarfism where shortened bone was found in limbs (Fig. 1a, b). Whole-mount skeletal staining by Alizarin Red and Alcian Blue showed that the length of forelimb and hindlimb was distinctly reduced in *Ror2* CKO mice (Fig. 1c, e) when compared with littermate controls (Fig. 1d, f).

### Impaired osteogenesis in neonatal *Ror2* CKO mice

**Fig. 2:**
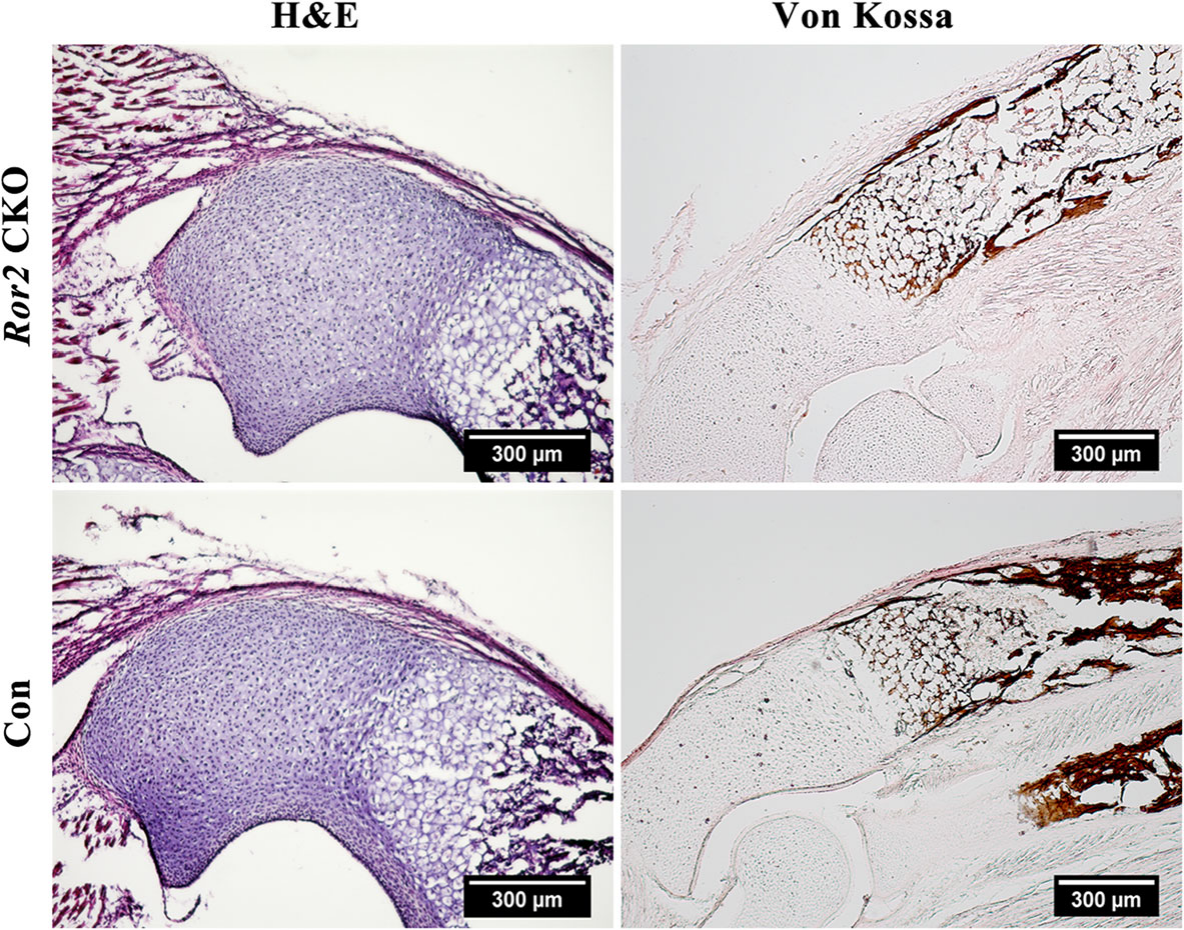
Histology staining of ulna proximal epiphysis presented an impaired osteogenesis in neonatal *Ror2* CKO mice (P0) compared with the littermate control (Con). Hematoxylin-eosin (H&E) and Von Kossa staining were used to observe the general histology and mineralized tissue, respectively. Scale bar = 300 μm. Compared with the control, mineralized tissue visualized in brown by Von Kossa staining was significantly decreased in *Ror2* CKO mice

**Fig. 3:**
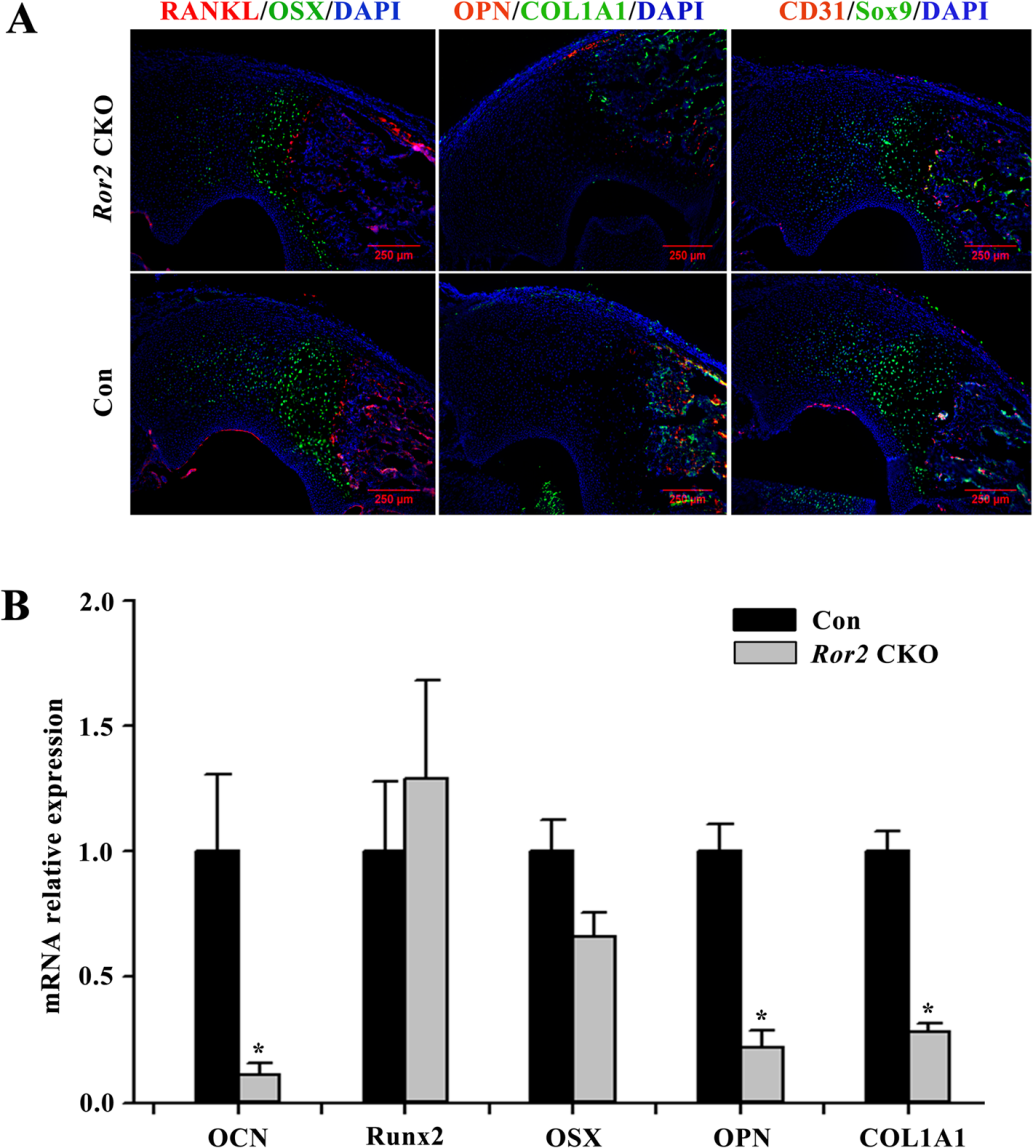
Reduced expression of osteogenic markers in neonatal *Ror2* CKO mice (P0) compared with the littermate control (Con). **a** Immunohistofluorescence staining of ulna proximal epiphysis showed that less OSX, OPN, COLlA1, and Sox9 were found in *Ror2* CKO samples. Scale bar = 250 μm. **b** RNAs were extracted from ulna proximal epiphysis. Quantitative real-time PCR revealed a significant decrease of mRNA expression of OCN, OPN, and COLlA1 in *Ror2* CKO compared with controls (*n* = 3, *P < 0.05 vs. Con)

Remarkably reduced mineralization in ulna proximal epiphysis was detected by Von Kossa staining in *Ror2* CKO mice (Fig. 2). IHF staining and qRT-PCR were performed with ulna proximal epiphysis of P0 mice. IHF staining showed that the osteogenic differentiation markers osterix (OSX), osteopontin (OPN), collagen I (COL1A1), and Sox9 were dramatically diminished in *Ror2* CKO mice (Fig. 3a). Consistent with IHF staining results, the mRNA expression of osteocalcin (OCN), OPN, and COL1A1 was significantly reduced in *Ror2* CKO mice compared with controls (*P* < 0.05; Fig. 3b).

### Knockdown of Ror2 in mBMSCs led to reduced osteogenic markers and Stat3 expression

**Fig. 4:**
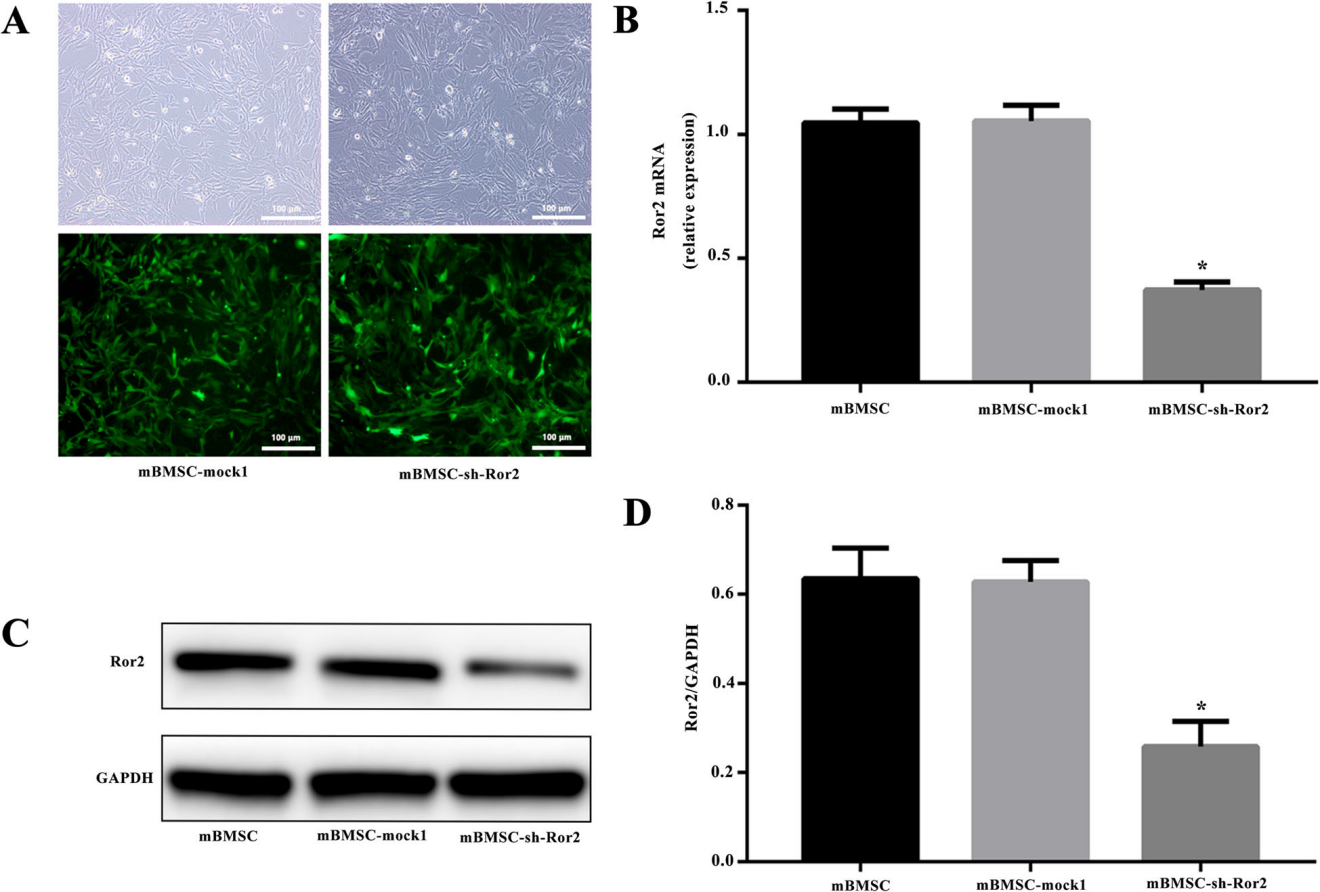
Knockdown of Ror2 in mBMSCs by the use of recombinant lentivirus vectors. **a** mBMSCs transfected with empty lentiviral vector (mBMSC-mock1) and shRNA targeting Ror2 (mBMSC-sh-Ror2) were observed with light microscopy (top) and fluorescent microscopy (below). Scale bar = 100 μm. **b** Quantitative real-time PCR analysis showed Ror2 mRNA expression in mBMSCs after transduction. **c**, **d** The expression of Ror2 protein in mBMSCs after transduction were evaluated using western blot analysis. *n* = 3, *P < 0.05 vs. mBMSC-mock1

**Fig. 5:**
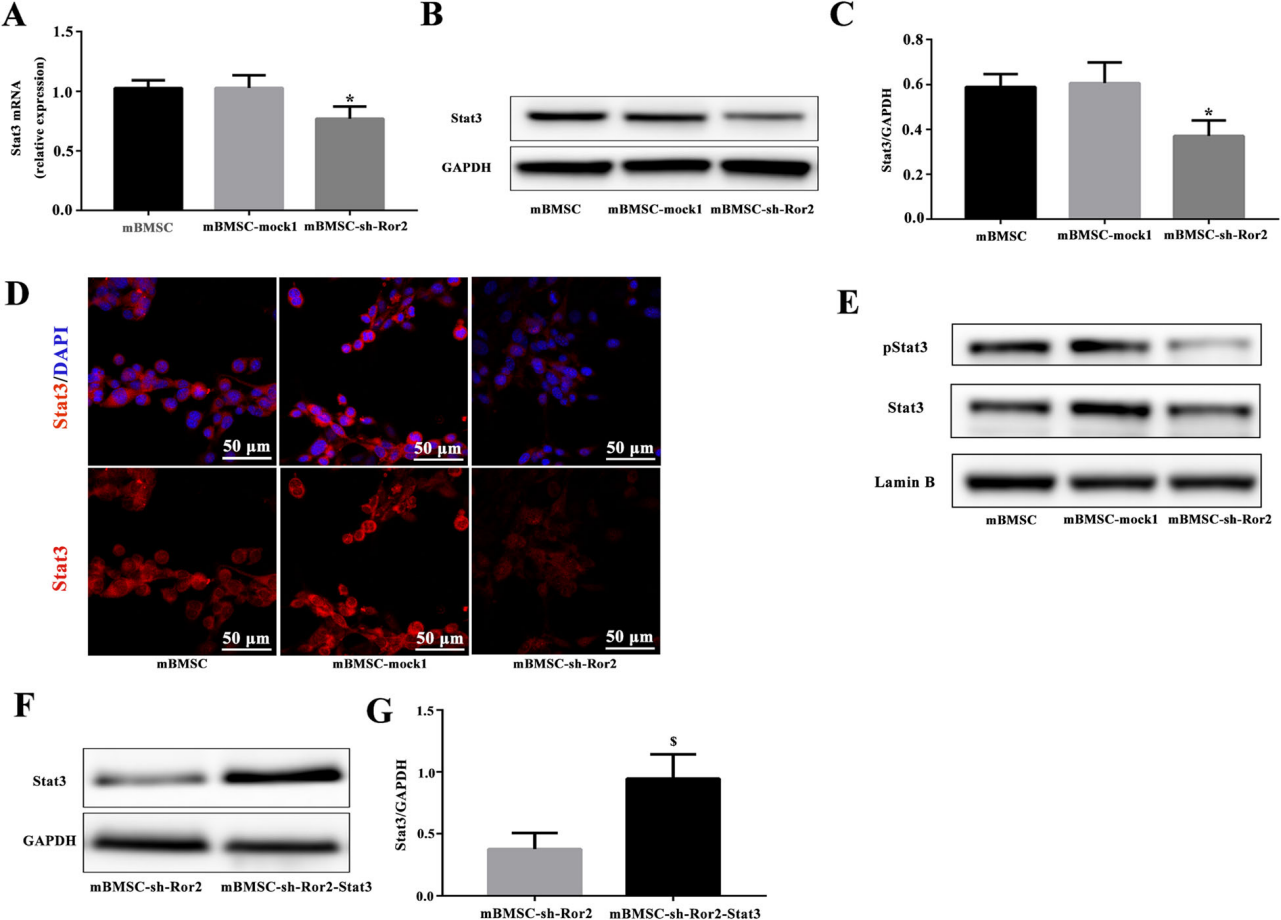
In Ror2-knockdown mBMSCs, the expression of Stat3 was decreased. **a**–**c** Quantitative real-time PCR (**a**) and western blot (**b**, **c**) analysis of the expression of Stat3 after osteogenic induction for 3 weeks. *n* = 3, *P < 0.05 vs. mBMSC-mock1. **d** Immunocytofluorescence staining showed the distribution of Stat3 after osteogenic induction for 24 h. Scale bar = 50 μm. **e** Western blot analysis revealed the expression of nuclear Stat3 and pStat3 after osteogenic induction for 24 h. **f**, **g** Overexpression of Stat3 in Ror2-knockdown mBMSCs. Stat3 protein expression was evaluated using western blot analysis. *n* = 3, ^$^*P* < 0.05 vs. mBMSC-sh-Ror2

**Fig. 6:**
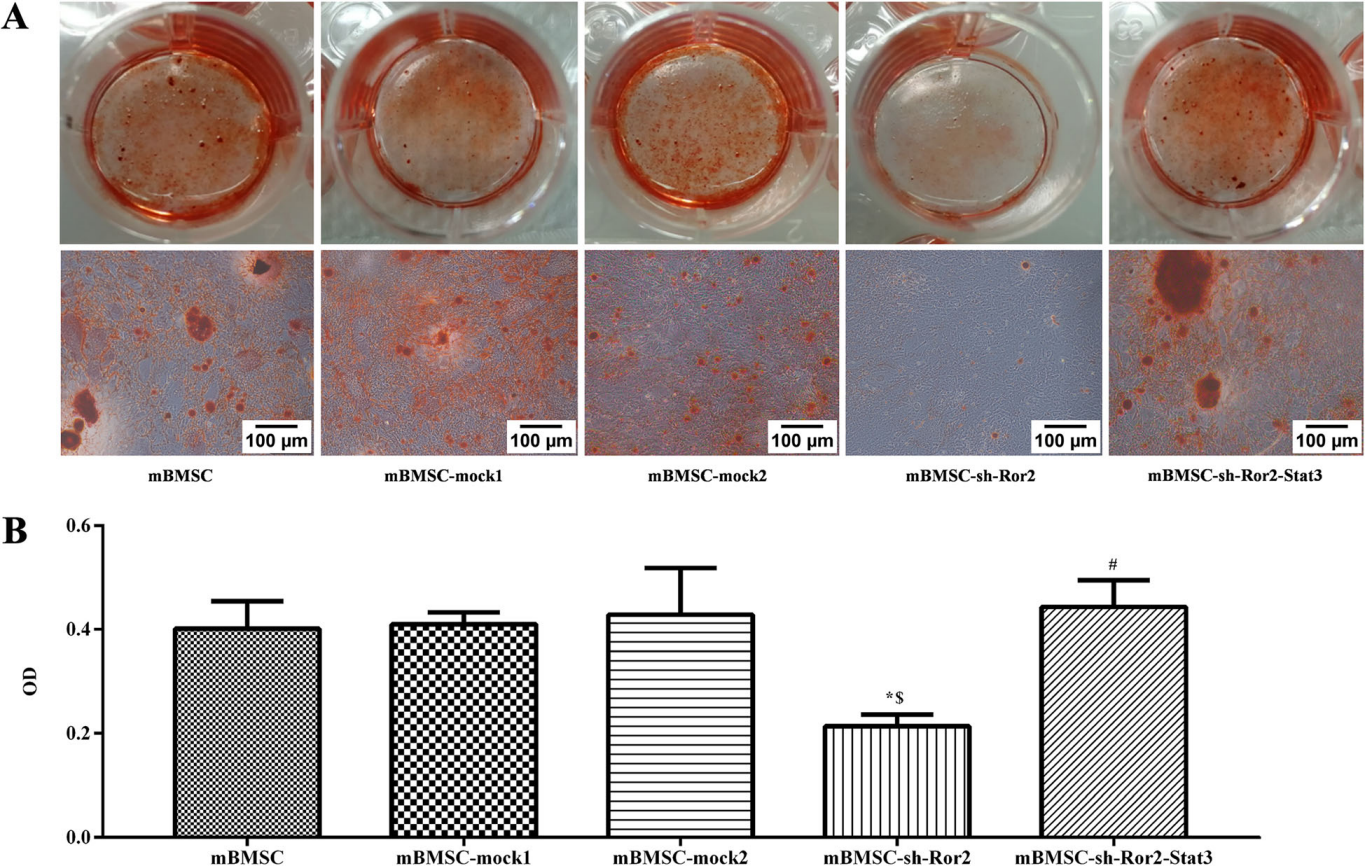
In response to osteogenic induction, the Ror2-knockdown mBMSCs showed decreased mineralization, which was rescued by overexpression of Stat3. **a** The cells were stained with Alizarin Red after osteogenic induction for 3 weeks. Scale bar = 100 μm. **b** Alizarin Red concentrations were determined by a quantitative destaining procedure using CPC. *n* = 3, *P < 0.05 vs. mBMSC-mock1; ^$^*P* < 0.05 vs. mBMSC-mock2; ^#^*P* < 0.05 vs. mBMSC-sh-Ror2

**Fig. 7:**
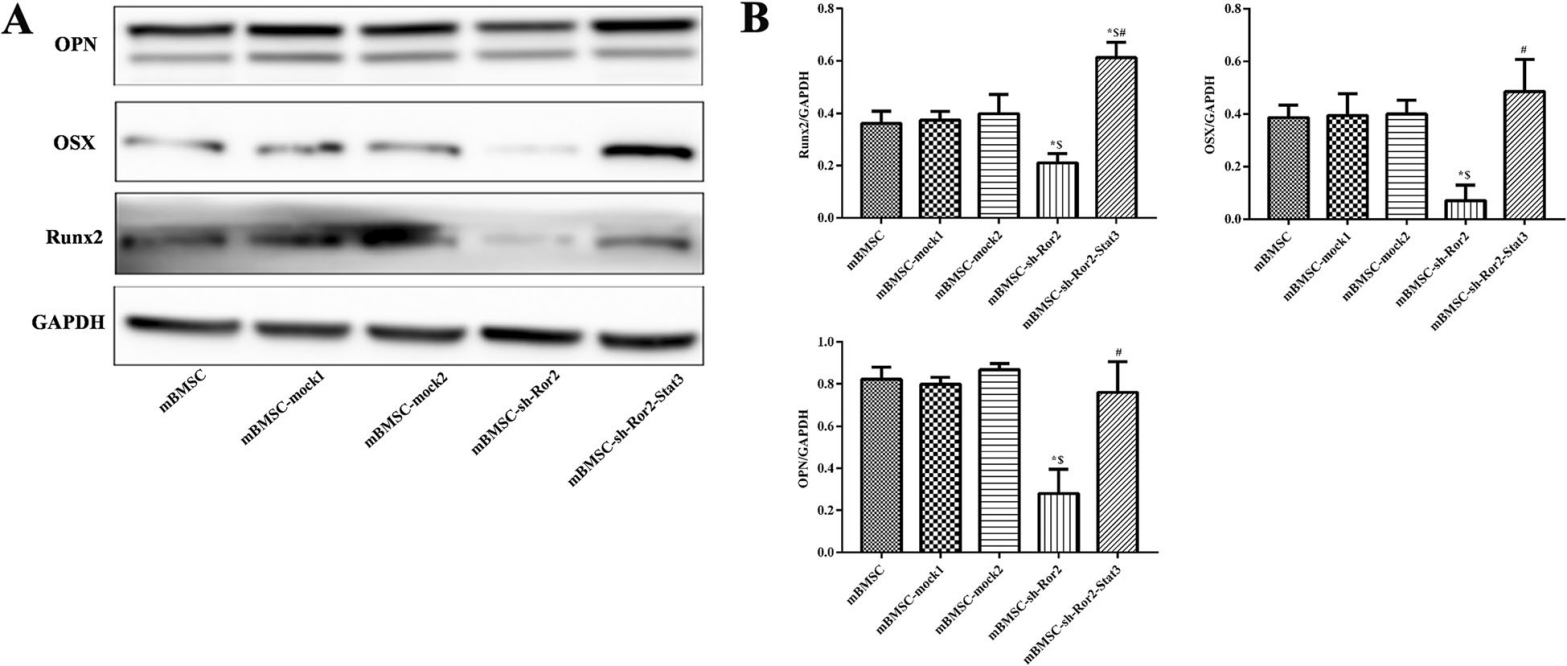
In response to osteogenic induction, the less expressed osteogenic makers in Ror2-knockdown mBMSCs were restored by overexpression of Stat3. **a**, **b** Western blot analysis of the expression of osteogenic specific markers after osteogenic induction for 3 weeks. *n* = 3, *P < 0.05 vs. mBMSC-mock1; ^$^*P* < 0.05 vs. mBMSC-mock2; ^#^*P* < 0.05 vs. mBMSC-sh-Ror2

Transfected with recombinant lentivirus vectors, the downregulation of Ror2 into mBMSCs was performed to investigate the effect of Ror2 on osteogenic differentiation from mBMSCs (Fig. 4a–d). After osteogenic induction for 3 weeks, less calcium deposit was observed in the mBMSC-sh-Ror2 cells when compared with mBMSC-mock1 cells examined by Alizarin Red staining (Fig. 6a) and quantitative Alizarin Red concentration measurements (Fig. 6b). The western blot results revealed that knockdown of Ror2 significantly suppressed the expression of Runx2, OSX, and OPN in mBMSCs in response to osteogenic induction (*P* < 0.05; Fig. 7a, b). No significant difference between mBMSC and mBMSC-mock1 was observed. Meanwhile, the expression of total Stat3 was significantly decreased in mBMSC-sh-Ror2 cells after osteogenic induction determined by qRT-PCR and western blot (*P* < 0.05; Fig. 5a–c).

Furthermore, ICF also showed that in comparison with mBMSC-mock1 cells, knockdown of Ror2 in mBMSCs caused a decrease of intracellular accumulation of Stat3 (Fig. 5d). At the same time, nuclear phosphorylated Stat3 (pStat3) was obviously reduced in mBMSC-sh-Ror2 cells after osteogenic induction for 24 h monitored by western blot (Fig. 5e).

### Reduced osteogenic differentiation in Ror2-knockdown mBMSCs was restored by overexpression of Stat3

In Ror2-knockdown mBMSCs, overexpression of Stat3 was conducted through utilizing recombinant lentivirus vectors (Fig. 5f, g). Alizarin Red staining demonstrated that similar amount of calcium deposits was observed in the mBMSC-sh-Ror2-Stat3 cells when compared with controls (mBMSC-mock1 and mBMSC-mock2) (Fig. 6a). Overexpression of Stat3 in mBMSC-sh-Ror2 cells caused a recovery of osteogenic ability to a level comparable to controls (mBMSC-mock1 and mBMSC-mock2) after 3 weeks of osteogenic induction, which was proven by the increased expression of osteogenic markers (Runx2, OSX, and OPN) (Fig. 7a, b). Thus, it was logical to speculate that Ror2 might facilitate osteogenic differentiation through Stat3.

## Discussion

In the present study, a significantly short limb phenotype was observed on *Ror2* CKO mice, where Ror2 was specifically knock out from limb bud mesenchyme and craniofacial mesenchyme. In comparison with the global Ror2 knockout mice which were neonatal dead due to forced respiration and severe cyanosis [11, 14], no difference of survival rate and lifespan was found between the *Ror2 CKO* mice and their littermate siblings. In the observation period of 4 postnatal weeks, the *Ror2 CKO* mice were alive with more conspicuous dwarfism over time. In our study, loss of Ror2 in limb bud mesenchyme resulted in considerably reduced mineralization in ulna proximal epiphysis. Our observation was in line with that of Takeuchi et al., which reported that a lack of calcification were observed in enchondral ossification centers when Ror2 was disrupted in mice [14]. The diminished expression of osteogenic markers in our study indicated that deletion of Ror2 in limb mesenchyme indeed impaired the ossification. As known, OCN and OPN, which are produced during bone formation and late in the mineralization process, play synergistic roles in determining bone size and shape [32]. The decreased expression of OCN and OPN in *Ror2* CKO mice indicated that Ror2 might be necessary for bone formation and morphology. In addition, during limb development, Sox9 was involved in the determination of both osteogenic and chondrogenic cell lineages. Neither mesenchymal condensations nor osteoblast lineage and its differentiation was observed in early limb buds of Sox9 depletion [33, 34]. Thus, the effect of Ror2 on bone formation might partly rely on regulating Sox9 signaling.

To gain insight into the cellular mechanisms of Ror2-mediated osteogenesis, we studied the effect of downregulation of Ror2 on osteogenic differentiation of mBMSCs in vitro. Available literature reported that the Ror2 expression was increased during early stages of osteoblast differentiation and was peaked in committed proliferating preosteoblasts, contributing to osteoblastogenesis [3, 10, 17]. Runx2 and OSX are expressed in preosteoblasts and play essential roles in the differentiation of preosteoblasts to immature osteoblasts [35]. In our study, when Ror2 was knockdown in mBMSCs, the osteogenic markers of mBMSCs, including Runx2, OSX, and OPN, were significantly decreased, which suggested the inhibition effect of Ror2 knockdown on mBMSCs’ osteogenesis. Our results were in accordance with previous reports, in which the overexpression of Ror2 in mesenchymal stem cells promoted the formation of mineralized extracellular matrix and expression of osteogenic transcription factors, whereas the downregulation of Ror2 resulted in the opposite [17, 36]. In addition, in preosteoblast cell line MC3T3-E1 and a mouse calvariae ex vivo organ culture model, Ror2 also strongly promoted matrix mineralization and increased bone formation [17, 37]. Collectively, it was proven that Ror2 played a promotive role in osteogenesis.

The expressions of Stats family have been found in bone tissues, which were believed to take part in activities of both osteoblasts and osteoclasts [24]. The increased bone mass and accelerated healing of bone fracture were observed in Stat1-deficient mice [38, 39]. The mechanism might be that Stat1 negatively regulated osteoblast differentiation by suppressing transcription of Runx2 and OSX. Stat4 and Stat6 were found involved in osteoclastogenesis and arthritis [40, 41]. Notably, Stat3 took part in a series of biological process of the bone, including cell growth, apoptosis, and motility. Furthermore, Stat3 was believed to be most important in osteoblasts and osteoclasts compared with other Stats [24]. Ror2 promoter region contained a GAS motif, which is speculated as a *cis* element for Stats [29, 42]. Inhibition of Stat3 in hADSCs reduced the ability of IL-6 to induce Ror2 mRNA expression and osteoblast-like differentiation and calcification [30]. These findings hinted a question that whether or not Stat3 was involved in the Ror2-mediated osteogenic differentiation of mBMSCs. In the current study, a significant decrease of Stat3 expression was observed in Ror2-knockdown mBMSCs in response to osteogenic induction at both mRNA and protein levels. Except cytokine receptors associated with JAKs, Stat3 can be activated by receptor tyrosine kinases and G protein-coupled receptors [43]. Upon ligand binding, Ror2 was found to form receptor complex with other components, including Src and CK1ε, then stimulates Stat3 activation, ultimately leading to cell invasion [44]. In our study, the overexpression of Stat3 in Ror2-knockdown mBMSCs rescued the decreased osteogenic differentiation of Ror2-knockdown mBMSCs. A possible explanation might be that the intracellular accumulation of Stat3 could promote the osteogenic gene transcription in mBMSCs cultured in osteogenic induction medium. However, whether or not Ror2 directly interacts with Stat3 in mBMSCs’ osteogenesis requires further study.

## Conclusions

Taken together, our study suggested a potential role and mechanism of Ror2 in mBMSCs’ osteogenic differentiation. Ror2 and Stat3 were essential for skeleton development by regulating mBMSCs’ osteogenesis and osteoblast differentiation. The impaired osteogenesis by knockdown of Ror2 was rescued by increased Stat3.

## Supplementary information

**Additional file 1: Figure S1.** The PCR results of genotyping *Ror2 CKO* mice. The protocol of genotyping was performed according to the Jackson Lab instructions. Lane 1: blank control (no DNA), Lane 2: negative control (DNA from wild-type mouse), Lane 3: Prrx1-cre; Ror2 ^c/+^, Lane 4: Prrx1-Cre; Ror2 ^c/c^(e.g. *Ror2 CKO*), Lane 5: Ror2 ^c/+^, Lane 6: Ror2 ^c/c^. **Figure S2.** The mBMSCs characterizations by flow cytometry.

## Data Availability

All datasets used and/or analyzed during the current study are available from the corresponding author on reasonable request.

## Abbreviations

Ror2: Receptor tyrosine kinase-like orphan receptor 2
Stat3: Signal transducer and activator of transcription 3
BMSCs: Bone marrow mesenchymal stem cells
OPN: Osteopontin
hADSCs: Human adipose tissue-derived mesenchymal stem cells
CKO: Conditional knockout
PCR: Polymerase chain reaction
IHF: Immunohistofluorescence
H&E: Hematoxylin-eosin
mBMSCs: Mouse bone marrow mesenchymal stem cells
qRT-PCR: Quantitative real-time polymerase chain reaction
FBS: Fetal bovine serum
BSA: Bovine serum albumin
ICF: Immunocytofluorescence
OSX: Osterix
COL1A1: Collagen I
OCN: Osteocalcin
Stats: Signal transducers and activators of transcription
