# Supplementary material for: Loss of receptor tyrosine kinase-like orphan receptor 2 impairs the osteogenesis of mBMSCs by inhibiting signal transducer and activator of transcription 3
Source: Stem Cell Res Ther. 2020 Mar 26;11:137. doi: 10.1186/s13287-020-01646-2 (PMC7098134; doi:10.1186/s13287-020-01646-2)
Supplement: Supplementary file 1 — Additional file 1: Figure S1. The PCR results of genotyping Ror2 CKO mice. The protocol of genotyping was performed according to the Jackson Lab instructions. Lane 1: blank control (no DNA), Lane 2: negative control (DNA from wild-type mouse), Lane 3: Prrx1-cre; Ror2 c/+, Lane 4: Prrx1-Cre; Ror2 c/c(e.g. Ror2 CKO), Lane 5: Ror2 c/+, Lane 6: Ror2 c/c. Figure S2. The mBMSCs characterizations by flow cytometry. [file 13287_2020_1646_MOESM1_ESM.docx]

**Supplemental Materials**

Figure S1 The PCR results of genotyping *Ror2 CKO* mice. The protocol of genotyping was performed according to the Jackson Lab instructions. Lane 1: blank control (no DNA), Lane 2: negative control (DNA from wild-type mouse), Lane 3: Prrx1-cre; Ror2 ^c/+^ , Lane 4: Prrx1-Cre; Ror2 ^c/c^(*e.g. Ror2 CKO*), Lane 5: Ror2 ^c/+^, Lane 6: Ror2 ^c/c^


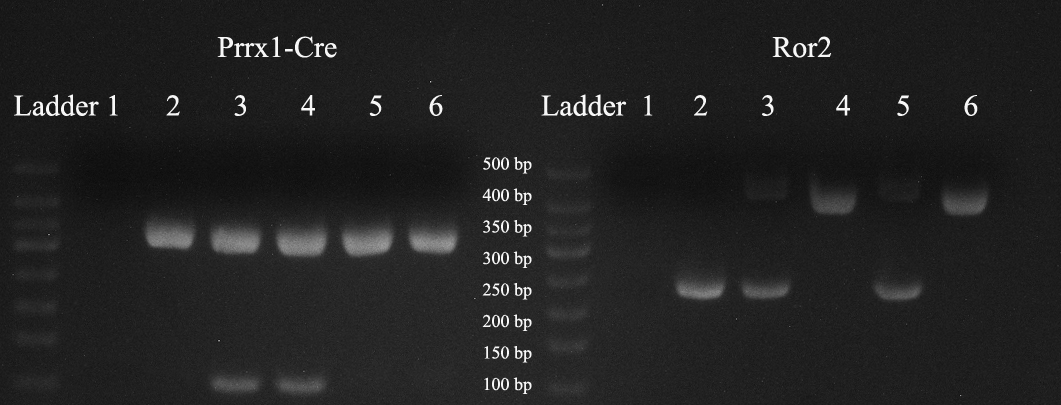


*(Prrx1-Cre : https://www2.jax.org/protocolsdb/f?p=116:5:0::NO:5:P5_MASTER_PROTOCOL_ID,P5_JRS_CODE:22392,005584) (Expected results of Prrx1-Cre: Cre~100 bp, control = 324 bp)*

*(Ror2: https://www2.jax.org/protocolsdb/f?p=116:5:0::NO:5:P5_MASTER_PROTOCOL_ID,P5_JRS_CODE:25306,018354) (Expected results of Ror2: Mutant = 438 bp, Heterozygote = 227 bp and 438 bp, Wild type = 227 bp)*

Figure S2 The mBMSCs characterizations by flow cytometry.

*wild type/Armenian Hamster IgG Isotype Control Antibody Anti-mouse/rat CD29 Antibody*

*Anti-mouse/human CD44 Antibody Anti-mouse CD117 Antibody*

*Anti-mouse Sca-1 Antibody Anti-mouse CD31 Antibody*
